# One world, one health: combating infectious diseases in the age of globalization

**DOI:** 10.1093/nsr/nwx047

**Published:** 2017-05-02

**Authors:** Jane Qiu

**Affiliations:** writes for NSR from Beijing, China.

## Abstract

There has been a resurgence of the H7N9 bird-flu virus in China since last winter, resulting in over 460 human infections—the largest number since the first outbreak in 2013—raising serious concerns about its further spread and the effectiveness of existing anti-viral drugs. This is just the latest example of the increasing threat from emerging infectious diseases. Due to a combination of factors related to farming practices, human behaviour, international travel, globalization and climate change, there has been a succession of such pandemics in recent years, such as Severe Acute Respiration Syndrome (SARS), Nipah, Middle East Respiratory Syndrome (MERS), Ebola and Zika, posing an unprecedented challenge to scientists and health workers worldwide.

In a forum organized by *National Science Review* at the World Life Science Conference last November, an international panel of scientists discussed the lessons that have learned from a string of pandemics in recent years, the importance of international collaboration and sharing research benefits more equitably, why there is an urgent need to move towards the one-health approach, and how China could play a leading role in the global effort to combat infectious diseases.

**Table:** 

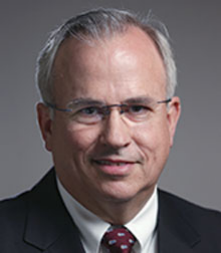	**Gregory Gray** Duke University, USA; Duke Kunshan University, China; Duke-NUS Medical School, Singapore	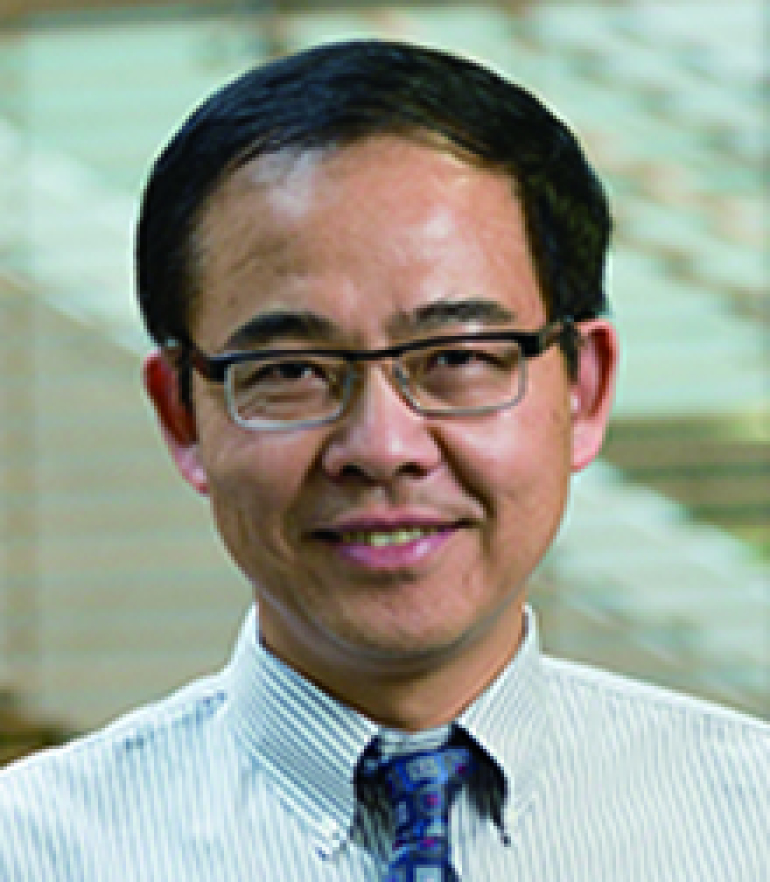	**Linfa Wang** Duke-NUS Medical School, Singapore
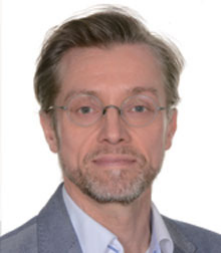	**Peter Horby** Center for Tropical Medicine and Global Health, University of Oxford, UK	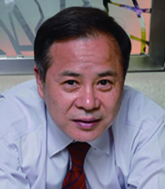	**Fujie Zhang** Capital Medical University, Beijing Titan Hospital, China
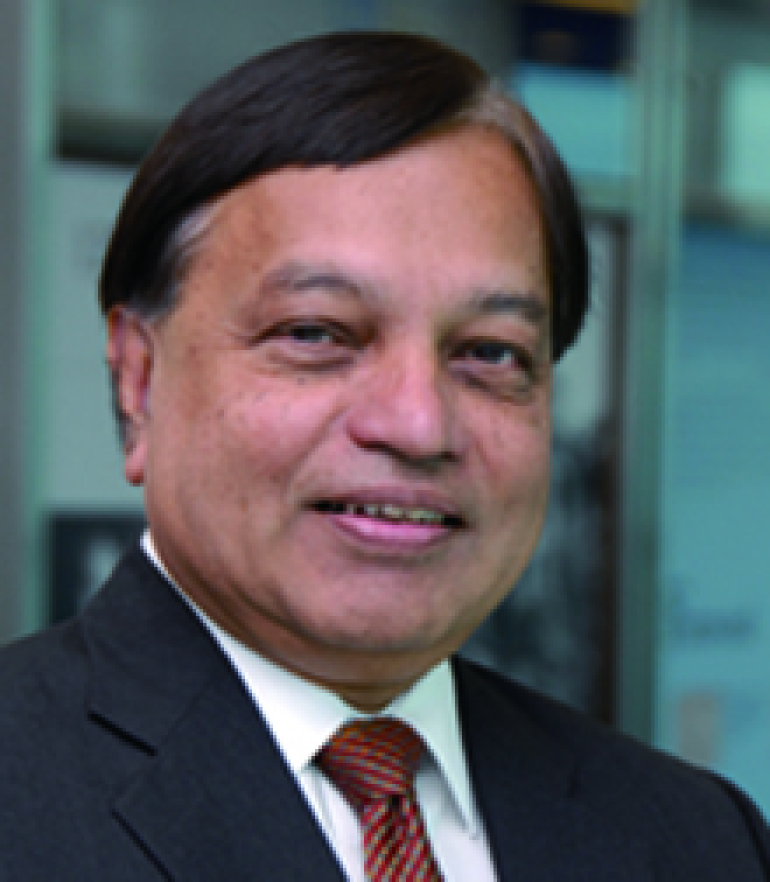	**Malik Peiris** University of Hong Kong, China	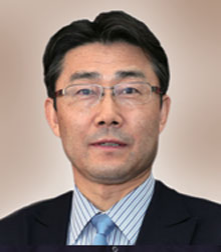	**George Fu Gao** Deputy Director General of the Chinese Centre for Disease Control and Prevention; Institute of Microbiology, Chinese Academy of Sciences, China

## ZIKA: A TALE OF TWO VIRUSES

**NSR:** Brazil has been hit quite badly by the Zika-virus epidemic since early 2015. What's the situation in Asia?

**Zhang:** The first recent case was detected in Singapore in June 2016. Since then there have been over 300 cases in Southeast Asia—especially Singapore, Thailand and Vietnam. In China, we have detected 22 cases.

**Peiris:** What's fascinating about the Asian cases is that the virus that caused the outbreak was not the Brazilian strain but those that have been around in the past 40 years.

**Wang:** This raises the question of whether we have always had Zika infection, which can be asymptomatic or cause mild symptoms such as fever and rash, making it difficult to precisely estimate the number of cases. Maybe some of the birth defects we have in Asia, such as microcephaly [a medical condition in which the brain does not develop properly resulting in a smaller than normal head] were caused by Zika.

The surveillance for Zika infection in most Asian countries remain rather superficial even after the major outbreak in Brazil, so you won’t be able to detect it unless you have a big cluster. Singapore raised the bar after the first cluster emerged. Then they found at least another eight clusters. If we perform that kind of stringent screening in other Asian countries, I can predict we will find many more clusters.

**NSR:** What have the sequencing studies told us about the epidemic?

**Wang:** They show that at least some clusters were caused by different lineages of the virus, which genetically were sufficiently different from one other. It's more likely that they had been around for quite a while—rather than one lineage giving rise to another within a few months or due to multiple introduction, a less likely scenario. This has important implications for the prospect of containing them. For this reason, the Singapore government is not making promises to completely eradicate the virus.

**Peiris:** A key is if the Asian strain of the Zika virus can also cause similar foetal damages to the Brazilian one.

**Wang:** That's on everybody's mind right now. It seems that the Asian strain is not as dangerous neurologically as the Brazilian one. But this is yet to be proved.

**Peiris:** Two sets of questions need to be addressed urgently. First, what's happening with Zika in Asia? Have these infections been around all this time? Or has the virus somehow changed in recent years and become more virulent or more able to cause epidemics? Second, what are the health consequences of the ‘Asian’ Zika infection? We should look into potential foetal abnormalities in a systematic way.

**Wang:** Indeed. We should see if there is a link between existing microcephaly case in Asia and viral infection. We could also do this prospectively. I'm involved in a study that involves 10 000 pregnant women, basically collecting blood samples at different stages of the pregnancy. We may not find a link between Zika and microcephaly, but we may uncover something else.

**Peiris:** A few pregnant women are among the confirmed cases in Singapore. It might be worthwhile following up those patients. Even though the number is small, this would give us some important information about whether the Asian virus acts differently or not. If none of them develop foetal damage, that would be rather reassuring.

It's the combination of big-scale farming industry and big-scale movements—of not only people but animals—that is responsible [for the increase in emerging infectious diseases].

—Malik Peiris

**Figure 1:**
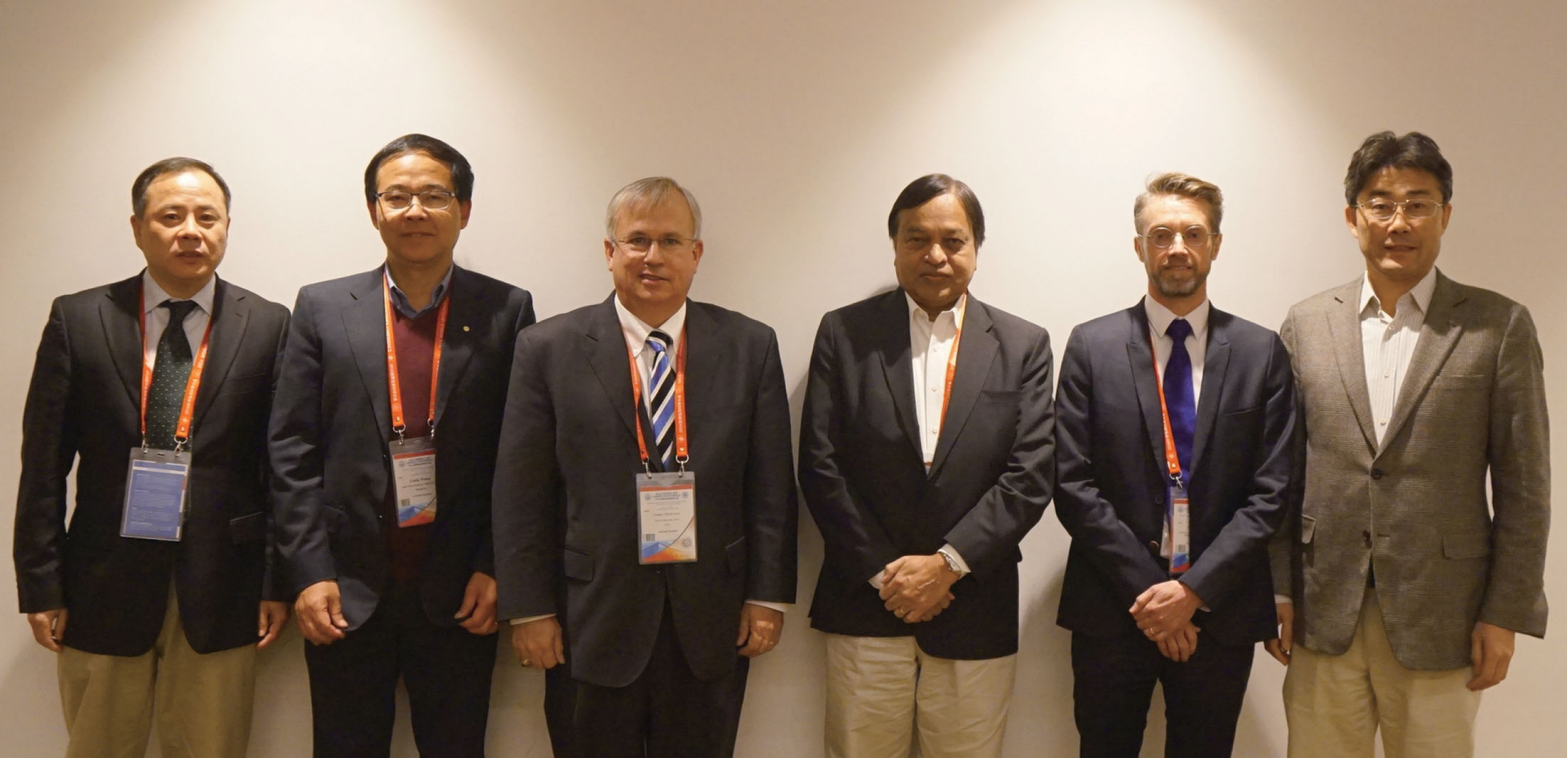
Six experts gathering together after the forum discussion. From left to right: Fujie Zhang, Linfa Wang, Gregory Gray, Malik Peiris, Peter Horby, George Fu Gao *(Photo: Xiaoling Yu, NSR)*.

## LESSONS LEARNED: CHINA AND BEYOND

**NSR:** We’ve had some serious pandemics in recent years, such as those caused by SARS and Ebola. What have we learned?

**Gao:** SARS was a game changer for China. At the beginning, people tried to cover it up, hoping it would just go away, but this made things much worse. China has learned a lot from the experience—in a hard way—and has changed its mentality and approach towards tackling infectious diseases. It now takes infectious diseases really seriously, and has a much more open and transparent policy. And it's keen to working with the international community not only in China but aboard, especially in resource-poor countries.

**Zhang:** China's public-health workers and officials have benefited tremendously from dealing with recent emergencies and from international collaboration and training workshops. This has afforded them a global-health mindset, more aware of infectious-disease risks from within China and abroad.

**Peiris:** Indeed. Huge changes have taken place in China since SARS. It's a definitive example of success stories. The early detection of MERS and Yellow Fever in the country are cases in point. I don't think this would have happened in pre-SARS times.

**Horby:** Ebola was also a step change for China. Now the country actively engages in global responses to emerging infectious diseases—providing on-the-ground support to foreign countries and doing excellent science that has important implications for determining response strategies.

**NSR:** How does China's public-health system work in preventing and control infectious diseases?

**Gao:** China's Infectious-Disease Prevention and Treatment Law is the cornerstone for combating infectious diseases. We have a network of Centre for Disease Control and Prevention (CDC) at the national, provincial, prefecture and county levels. They are capable of diagnosing all the infectious diseases that are known to exist worldwide regardless of their prevalence in China, and are responsible for carrying out systematic vaccination, surveillance, testing and reporting. We’ve also set up dedicated infectious-disease hospitals in all large and medium-sized cities.

Human health and animal health are intimately related and the one-health approach is the only way to tackle emerging infectious diseases in a globalized world.

—Gregory Gray

The law requires that incidents of 39 specified infectious diseases must be reported within 24 hours of confirmation and those that are new to the country or have been announced to be eliminated must be reported within 2 hours. Moreover, there is a multi-sectorial coordination system related to acute infectious diseases—including relevant ministries under the State Council, led by the National Health and Family Planning Commission—and compulsory quarantine can be quickly implemented when suspected cases are found.

**Zhang:** In March 2016, our hospital confirmed China's first imported Yellow Fever incidents 44 hours after the patient's return from Angola. The patient was initially suspected of having Dengue Fever, but DNA sequencing by our hospital and the national CDC showed that it was Yellow Fever. This prompt confirmation was a result of improved personnel and technological diagnostic capacities.

**NSR:** The Ebola pandemic between late 2013 and early 2016 in West Africa was the worse incidence in recent years, causing over 11 300 deaths. Why was it so bad?

**Gao:** The key to preventing and controlling acute infectious diseases is the ‘four-early strategy’: early discovery, early diagnosis, early quarantine, and early intervention. Regarding Ebola, the main problem lies in the failure in early intervention. The three West Africa countries did not pay much attention to this matter at the beginning, and it became really difficult to contain the infection once it had spread. They didn't have the diagnostic capability or enough healthcare workers. There was also a lack of medicine and basic nutrition or food for sustaining the patients.

And the help came too late and too feeble. The Pasteur Institute first confirmed the cause to be Ebola in March 2014. The World Health Organization did not openly announce the seriousness of the epidemic until August 2014. Even then, most countries only reacted at the level of discussion and did not send their medical staff to the frontline to curtail the disease. There is an immediate need to train healthcare workers on the ground and help them with the diagnosis, quarantine and intervention when an outbreak occurs.

**NSR:** What was China's role in combating Ebola?

**Gao:** The aids provided by the Chinese government amounts to 750 million RMB. I was in Sierra Leone from September 2014 to November 2014. We helped build a BSL-3 biosafety lab in Sierra Leone and a hospital in Nigeria. We trained public-health staff there, and taught in local schools and communities about what the disease was about and key prevention and control strategies.

The Ebola pandemic really hits home the fact that infectious diseases are a global issue that requires collaborative efforts from many countries. As the lack of adequate public-health provisions in many developing countries impedes their ability to tackle acute infectious diseases, developed countries must provide rapid and effective responses when an epidemic occurs.

## ONE WORLD, ONE HEALTH

**NSR:** Are we seeing an increasing trend of emerging infectious diseases in recent decades?

**Horby:** One study estimated that around 300 new infectious diseases emerged over the past 60 years. We have to be cautious in interpreting these estimates because our ability to diagnose those diseases have also increased dramatically during that time. So it's much easier to detect infections that may have been around for a long time but haven't previously been recognized. But even after this is taken into consideration, it looks like there is still an increase.

**Gao:** Indeed. We are definitely seeing more infectious diseases. There are many drivers for this, such as human behaviour changes, ecological changes, climate change and globalization. Now people travel everywhere. Once a pathogen emerges, it can spread rapidly—just an overnight flight from Africa to China—posing a big challenge to combating infectious diseases.

**Zhang:** As China's foreign-trade volume increases dramatically, we’ve also had more infectious diseases—such as Zika, Yellow Fever, and Rift Valley Fever—imported from Africa and Latin America. It has tightened its public-health measures, especially in border controls.

**Peiris:** The industrialization of farming is definitely a major factor. This is certainly the case in China, where the pressure for food production is intense. We’ve seen a proliferation of large livestock farms that have the capacity of raising tens of thousands animals of the same species—some of which are probably naive animals and haven't been exposed to any pathogens. This can sustain a much greater variety of viruses and provide more opportunities to give rise to new ones that can infect humans.

**Gray:** And livestock get transported across the country and their products around the world. Compounded with a sharp increase in international travel, we have this perfect storm in the sense that viruses carried by animals could easily cause a major global epidemic. We know that 70% of human infectious agents come from animals.

**Peiris:** A good example is the H7N9 bird flu. We know that chicken got the virus from wild birds in eastern China. But the spread of infection took place purely through poultry trade. This type of spread would never have happened half a century ago. It's the combination of big-scale farming industry and big-scale movements—of not only humans but animals—that is responsible.

**Gray:** That's why human health and animal health are intimately related, and the one-health approach—the collaborative efforts of multiple disciplines, which work locally, nationally and internationally to attain optimal health for people, animals and our environment—is the only way to tackle emerging infectious diseases in a globalized world.

## THE ROLE OF INTERNATIONAL COLLABORATION

**NSR:** What's the role of international collaboration in combating infectious diseases?

**Gao:** International collaboration is absolutely important because pathogens have no respect for national borders. This is especially important in a globalized world and with the level of international travel we have now. The challenges, however, are immense—from sharing specimens, political sensitivities to international tourism.

**Horby:** Indeed. Infectious diseases are a global-health threat. They can spread rapidly, so we need to have good evidence to properly respond to it. In an outbreak, we normally see small clusters at the beginning, so we need to work across countries to get enough cases to make the results statistically meaningful. The Zika epidemic is a good example of such cohort studies. Moreover, to understand whether the Asian and Brazilian strains have different heath impact, we need to use the same definitions of things like microcephaly and gestation, and to standardize procedures so results can be compared.

**Wang:** Relationship building has to happen on a regular basis—with or without an outbreak. We can't say ‘let's have a conference and build relationship because we have an outbreak’. It would be too late. I always like to use the terms of ‘peace time’ versus ‘war time’. We have to prepare for the war and get the preparedness level up during peace time.

**Zhang:** Here at Beijing Tiantan Hospital, we work closely with organizations such as the US CDC, World Health Organization (WHO), and the Global AIDS Programme. We are discussing with our American colleagues about a new project between our hospital and the US CDC on emerging infectious diseases. We are particularly strong in diagnostics and have a large collection of patient samples for scientific research.

**NSR:** How could international collaboration help build capacity in developing countries?

**Horby:** Traditionally, a small number of people from developing countries get trained in the West. But often, they don't go back to their home countries. When they do, they often find it difficult to apply their skills there because things work differently. For the past 25 years, the Oxford University Clinical Research Unit—based within the Ho Chi Minh City Hospital for Tropical Diseases and under the direction of Vietnam's Ministry of Health—has provided an alternative model, effectively moving training opportunities to the developing world.

This has allowed people to be trained on problems that are locally relevant and without having to leaven their families behind. It also gives them a career structure afterwards, which is really important and has to be part of the capacity-building process. Equally importantly is the fact that international collaborators are permanently based in low-income settings and have helped not only improve health systems but build an excellent centre for clinical research that attracts world-class researchers (because of its proximity to the frontier of infectious diseases with easy access to the problem and patients). This has built a very strong level of trust and relationship.

International collaboration is absolutely important because pathogens have no respect for national borders.

—George Fu Gao

**Gray:** Duke University is very global-health-oriented. Its Global Health Institute partners with institutions in more than 60 countries. The projects, mainly driven by individual faculty, involve numerous types of medical problems, including infectious diseases, and engage a wide range of public sectors.

## SHARING SPECIMEN AND RESEARCH BENEFITS

**NSR:** International collaboration is not always plain sailing. During the 2007 H5N1 bird-flu outbreak, Indonesia refused to share virus samples and threatened to shut down a US research unit in Jakarta. What happened?

**Peiris:** What happened was that one of the commercial companies developed a vaccine based on a virus from Indonesia. That vaccine was, however, too expensive for use in Indonesia. The Indonesian government couldn't afford it and, understandably, got very upset, resulting in a breakdown in the sample-sharing process. WHO went through many years of negotiation and came up with this Pandemic Influenza Preparedness (PIP) Programme, which entails that countries that share virus samples would get benefits through WHO in a number of ways, such as vaccines and anti-viral drugs and building the capacity to develop and manufacture vaccines locally.

When the 2009 pandemic emerged, WHO was able to distribute vaccines and anti-viral drugs to developing countries around the world. It was not enough for everybody, of course, but at least there was some level of benefit sharing. PIP hasn't totally solved the problem—and it applies only to influenza—but it's a start.

**Horby:** Indonesia's objection to share H5N1 samples has had a positive impact because it forced people to think about equitable sharing of research benefits. The WHO framework is the way forward—even though it currently deals with only influenza.

**Peiris:** It's really a dilemma. On one hand, you need to share materials when you have an unusual outbreaks. On the other, you need to protect the intellectual benefits that come with it.

**Gray:** Intellectual-property rights are a major stumbling block to sharing data and specimens. Indonesia is a case in point. We also saw restrictions from Saudi Arabia regarding sharing MERS coronavirus specimens. They were worried about losing out if they shipped specimen to a research group, which then licensed the virus or a related product and marketed it in diagnostics or vaccines. We need to think about these economic issues. In recent years scientists have occasionally patented viruses, which can seriously impede the sharing of data and specimens. The WHO is trying to mitigate this, but I don't see an easy solution.

Relationship building has to happen on a regular basis—with or without an outbreak.

—Linfa Wang

**Wang:** I think there are three levels of stumbling blocks: scientists don't want to share, countries don't want to share, and, even if they do, there are operational issues, such as importation–exportation restrictions.

**Gao:** It's really in everybody's interest to share. But there has to be a large degree of trust, which takes a long time to build. There has to be real willingness, particularly from the developed world, to share the benefits of research equitably between all parties.

**Zhang:** Sharing data and specimens is indeed a very sensitive issue in China. It's even quite difficult to share between departments within an institution because data and specimen mean publications—which, in turn, mean promotion and financial gains. So each department sets up its own database and guards it very closely.

I think the National AIDS Control and Prevention Centre within the China CDC has set up a good model for information sharing. It integrates different databases—such as the HIV epidemiology database, the anti-retroviral therapy database, and the maximum maintenance clinical database—so we can link different databases and track development. This is a government-led initiative within the CDC, probably the only way to improve the situation for other infectious diseases.

**NSR:** How about sharing specimen with international institutions?

**Zhang:** It's very difficult because China has very strict rules about exporting materials that contain human genetic information—though there are procedures in place to get government approval under extraordinary circumstances. In our collaboration with foreign scientists, all the lab work has to be done in Beijing.

**Peiris:** During the 2013 H7N9 bird-flu outbreak, the China CDC shared virus sequences very rapidly and later also shared viral samples. The sharing of the virus had to go through procedures and it took 3–4 weeks.

**Wang:** That's considered as very good already, but it's simply not good enough. Influenza outbreaks is certainly leading the way in terms of sharing specimen. You would not have that with other infectious diseases. It's really a major challenge.

**Gray:** Many of my US colleagues find it very difficult to collaborate with China because its strict rules about exporting tissue samples and live organisms. This has greatly inhibited the partnering between US and Chinese scientists because it's more of a one-way street. So one thing China could think of doing is to be more open and sharing in ways that it doesn't harm its economy or the interests of Chinese scientists.

**Peiris:** I can understand that when China wasn't so well developed in scientific research, it is worried that it would lose out when specimens were shared and someone else in the West ended up publishing the work. The situation has changed and China now has very high levels of science. It's probably time to relax its defensive strategy a bit about sharing—because unless you share, you can't gain the benefits of sharing and move forward.

## MAIN CHALLENGES

**NSR:** What are other challenges in combating infectious diseases?

**Horby:** Infectious diseases are a massive burden to our society. Take influenza as an example: 10–20% of global population are infected, including 3–5 million severe cases, resulting in an estimated half a million deaths. But there is still a major mismatch between the damage they cause and funding at government and international levels.

**Wang:** To persuade governments to invest more in infectious diseases remains a big challenge. While it can be really bad when there is an epidemic, it comes and goes. And when it's gone, people forget how bad it was. It's much easier to get funding for diseases such as cancer, cardiovascular diseases and neurological diseases. There needs to be studies to show in real economic terms the impact of infectious diseases. Governments need to see if they invest one dollar how much they can save.

**Peiris:** There is certainly an economic case to be made for having an effective disease surveillance and control capacities. One wonders how much it would have cost China if it didn't detect the recent cases of MERS or Yellow Fever so early, but putting a number on the economic impact is challenging.

**Horby:** Zika is a good example of the lack of preparedness during ‘peace time’. The virus has been around in the past 40 years. Now there are major uncertainties about the impact of prolonged infection on the nervous and reproductive systems. If we had investigated this virus in the past, we would have been able to answer those questions.

**Peiris:** But there are so many infectious pathogens. How do we prioritize which ones to focus on?

**Horby:** It's tricky. WHO and others are trying to prioritize which ones to invest in their R&D frameworks. There are two approaches. On one hand, you can try to predict which ones are likely to be dangerous, such as influenza and coronaviruses. This would take out a big chunk of risks, though there will always be those that you'd never to able to predict. On the other, you can invest in generic platforms, such as diagnostic platforms or improved trial designs.

**Wang:** Another challenge is early reporting. I’ve come across incidences in which an emerging epidemic wasn't made public more than six months after the first case was detected. The governments were reluctant to let the outside world to know for fear of impact on trade and tourism.

**Peiris:** There can be unjustified penalties associated with good behaviour in early reporting, leading to restrictions in trade or travel. We have to somehow disentangle this. Otherwise it would be a lose–lose situation.

**Gao:** From research point of view, a priority is to understand the mechanisms that underlie cross-species transmission. Insights into whether and how a virus affecting one species become infectious in another is critical for developing effective preventive measures. The Chinese Academy of Sciences has recently established the Centre for Influenza Research and Early-warning. One of our goals is to solve the problem of cross-species transmission through a multidisciplinary approach, which is in line with the one-health philosophy.

Regarding transmission of viruses by migration birds, for instance, zoologists would examine the migratory behaviour of birds, virologists and molecular biologists would study how avian viruses are capable of infecting human cells, and the China CDC—which has established a national network for monitoring infectious diseases throughout the country since the 2003 SARS pandemic—would provide timely information on disease incidence in various regions. Integrating multiple lines of information will allow us to understand the nature of infection, and whether and how viruses spread cross-species.

**Horby:** There is also an urgent need to speed up clinical trials, so they could respond to infectious-disease outbreaks much more quickly. A study by Cancer Research UK found that it took an average of 621 days (or 20 months) to recruit the first patient after funding was agreed—much longer than most epidemics.

**Gao:** Governments should take a more proactive initiative towards developing vaccines for infectious disease. It's an important public-health issues and should not solely rely on the pharmaceutical industry.

**Horby:** Another issue is the lack of research on health services, which concerns how to correctly diagnose infectious diseases and properly manage patients at different levels of the healthcare sectors. This type of research is not sexy and doesn't give you papers in high-impact journals. But it's incredibly important.

**Wang:** In Singapore, this is taken so seriously that they set aside funds dedicated to this type of healthcare research. Most people carrying out such research are economists and social scientists, who don't have Nature or Science papers. But they are very knowledgeable and established in their own disciplines and are doing very important work.

**Horby:** We should take a broad perspective of what science is about. It should reflect a whole spectrum, not just lab work. There is an urgent need to incorporate social science and behavioural science into studies of infectious diseases.

**Gray:** Equally important is to communicate freely and rapidly findings of new infectious diseases. This could be hampered by the desire to publish results in high-impact journals, which tend to have very strict rules about not releasing the results before submission.

**Peiris:** In the case of Zika, there are now websites for people to communicate pre-publication information, which shouldn't compromise their chance to publish in high-impact journals.

**Horby:** Some medical journals have indeed made statements that posting preliminary data on a website would not jeopardize publication. But when it comes to submission, they would say, well, it's not very interesting because we know that already. I'm yet to be convinced that it works in practice. Journals also need to committee to publishing faster when papers on infectious diseases are concerned.

Indonesia's objection to share H5N1 virus has had a positive impact because it forced people to think about equitable sharing of research benefits.

—Peter Horby

New technologies have a huge potential in monitoring infectious-disease outbreaks in real time. For instance, a cheap and palm-sized sequencing device called MinION developed by Oxford Nanopore really hit the ground during the Ebola outbreak. It allows quick genetic sequencing of infectious agents and allows us to monitor mutations and track transmission chains, which is critical for stamping out the epidemic.

## STEPPING UP THE GAME

**NSR:** What else does China need to do to step up its effort in tackling infectious diseases?

**Gao:** China should invest more resources. The China CDC has a total staff of 2267 for 1.4 billion population—compared to 30 000 staff at the US CDC for 0.3 billion population. More importantly, the work of China CDC is expanding along with the One-belt, One-road Initiative [a development strategy that focus on connectivity and cooperation between China and other Eurasian countries] and South-South Cooperation, for which China will have a more important role in combating infectious diseases.

**Zhang:** Indeed. The number one challenge for China is that, as a developing country, it's still poor in medical resources, especially cutting-edge facilities and well-trained staff. While this may not be obvious in big cities and provincial capitals, it's an entirely different picture if you go down the chain. And healthcare systems—in terms of both public health and clinical care—are particularly under-developed in rural regions, where new infectious diseases are more likely to emerge.

**Gray:** The way forward might be to identify minimal standards of healthcare services, put the resources there and try to make sure they are equitable.

**Peiris:** I'm actually rather impressed with the standard of the provincial CDC in Guangdong, which is very active and proactive. But then it's a big and rich province that is prone to infectious-disease outbreaks. Its proximity to Hong Kong also means that it has more access to international expertise. Things may be quite different in other provinces. I think augmenting the whole network is very important—because the weakest link could jeopardize the entire endeavour.

It's even quite difficult to share [data and specimen] between departments within an institution [in China] because data and specimen mean publications, promotion and financial gains.

—Fujie Zhang

**Zhang:** Long-term capacity building of public-health institutions at all levels across China remains key to combating infectious diseases. This involves surveillance, the quality of medical treatment, scientific and technological competence, as well as the ability to rapidly respond to an outbreak. The diagnostic capacity of CDC laboratories is key to manage epidemics.

**Gray:** It seems that China's biggest cities, such as Beijing, Shanghai and Guanzhou, have excellent public-health provisions. They could be training centres for rural regions. Meanwhile, you could also follow the Oxford model within China, creating financial and professional incentives for people to work at provincial and county levels.

**Horby:** China is in a unique situation because it has a broad spectrum of capabilities—from extremely advanced to extremely under-developed. This places it in a good position not only to help capability building in developing countries but to mentor developed countries about how to apply research and technologies to resource-poor settings.

**Gray:** The US CDC runs an Epidemic Intelligence Service programme, in which professionals receive epidemiological training and sometimes are matched to serve in more rural US areas. Even though working in a rural setting, they are still accountable to the national CDC. Maybe it's a model to think about. This could raise the standard of vaccination, surveillance and infection control.

Another issue, which is not unique to China, is the urgent need to move towards the one-health approach. This calls for much closer cooperation and collaboration between the human-health and animal-health sectors—both on the government and academic levels. Meanwhile, China should not only bridge the gap between urban and rural health but pay more attention to what's going on in neighbouring countries.

**Peiris:** While the primary motive of the One-belt, One-road Initiative is economic, this could be an ideal platform for boosting international collaboration in combating infectious diseases. One way to go about it could be to have regular scientific meetings that bring together scientists, healthcare workers and public-health officials from Asian countries. You can't force people to work together. But once they get to know each other through this kind of gatherings, information begins to flow and collaboration becomes a natural outcome—a much more effective approach than top-down efforts.

**Wang:** This is a very nice suggestion. The meeting should be regular, every two years or so, and have a brand name. It would be the meeting to go to for everybody working on infectious diseases, effectively establishing an on-the-ground working relation in a non-governmental way. Ideally, there should also be a pan-Asia CDC, where governments and scientists can really work without borders.

China CDC might want to consider ways to further boost international collaboration. It could establish an international centre of excellence within China CDC; it could have grants dedicated to projects that require Chinese and foreign institutions working together; it could also set up exchange-scholar schemes with CDCs in other countries.

**Horby:** China could be a role model by being a global leader and an open country in the scientific sense. Ebola is a good example where China reached out and used its scientific and clinical expertise on a global platform. There is still room for China to engage even more proactively internationally, especially in terms of sharing data and specimens. There are clear benefits of openness and transparency. This would allow closer collaboration with China, which many people in West are very keen to see because there are great scientists here and great questions to be answered.

